# Case report: Identification of a novel HNRNPC::RARG fusion in acute promyelocytic leukemia lacking RARA rearrangement

**DOI:** 10.3389/fonc.2022.1028651

**Published:** 2023-01-09

**Authors:** Wenjing Ding, Guangyang Weng, Zheng Wang, Yusha Guo, Man Wang, Hongjie Shen, Suning Chen, Xin Du, Lijun Wen

**Affiliations:** ^1^ Jiangsu Institute of Hematology, Key Laboratory of Thrombosis and Hemostasis of Ministry of Health, the First Affiliated Hospital of Soochow University, Soochow University, Suzhou, China; ^2^ Institute of Blood and Marrow Transplantation, Collaborative Innovation Center of Hematology, Soochow University, Suzhou, China; ^3^ Department of Hematology and Shenzhen Bone Marrow Transplantation Public Service Platform, Shenzhen Second People’s Hospital, The First Affiliated Hospital of Shenzhen University, Shenzhen University School of Medicine, Shenzhen, China; ^4^ Suzhou Jsuniwell Medical Laboratory, Suzhou, China

**Keywords:** acute promyelocytic leukemia, translocation, RARG, HNRNPC, Venetoclax

## Abstract

Acute promyelocytic leukemia (APL) is a special subtype of acute myeloid leukemia (AML), 95% patients have PML-RARA fusion gene as a result of a reciprocal chromosomal translocation t(15;17)(q22; q21). The retinoic acid receptors (RARs) belong to nuclear hormone receptors which modulate the transcription of DNA elements. RARs have three isoforms: retinoic acid receptor alpha (RARA), retinoic acid receptor beta (RARB) and retinoic acid receptor gamma (RARG). In this study, we describe the experimental results of a case with HNRNPC::RARG gene transcript with morphologic and immunophenotypic features similar to APL, including bone marrow morphology and immunophenotype, which showed poor response to ATO and chemotherapy. Then the patient achieved remission under the combination of BCL-2 inhibitor (Venetoclax) and standard 7 + 3 chemotherapy in second induction chemotherapy. The treatment in this case demonstrated effective response to Venetoclax, which suggested its possible role for the patient with acute promyelocytic-like leukemias (APLL).

## Introdution

Acute promyelocytic leukemia (APL) is a special subtype of acute myeloid leukemia (AML), in which 95% patients have the PML::RARA (promyelocytic leukemia-retinoic acid receptor alpha) fusion gene as a result of a reciprocal chromosomal translocation t(15;17)(q24;q21). The retinoic acid receptors (RARs) belong to nuclear hormone receptors which modulate the transcription of DNA elements, including three isoforms: retinoic acid receptor alpha (RARA), retinoic acid receptor beta (RARB) and retinoic acid receptor gamma (RARG). The majority of APL is characterized by a t(15;17) translocation resulting in the PML::RARA fusion gene, which is sensitive to therapy with all-trans retinoic acid (ATRA) (1). However,in few cases of APL, we failed to identify the PML::RARA fusion gene, while instead found some other partner genes with RARA, such as the promyelocytic leukemia zinc finger (PLZF) on chromosome 11q13; nucleophosmin 1 (NPM1) on chromosome 5q35; the nuclear mitotic apparatus (NuMA) on chromosome 11q13; the signal transducer and activator of transcription 5 (Stat5b) on chromosome 17q11; the heterogeneous nuclear ribonucleoprotein C (HNRNPC) on chromosome 14q11 (2–6). As a member of the nuclear receptor family, in addition to retinoic acid receptor alpha (RARA), the retinoic acid receptor (RAR) has other two isoforms: retinoic acid receptor beta (RARB) and retinoic acid receptor gamma (RARG) (7). In recent years, rearrangements involving RARB and RARG in resembling APL were identified one after another, including the fusion transcripts ransducin (beta)-like 1X-linke receptor 1 (TBL1XR1)::RARB, PML::RARG, nucleoporin 98 (NUP98)::RARG, and cleavage and polyadenylation specific factor 6 (CPSF6)::RARG (8–11). In the present study, we report a novel variant APL patient lacking the typical PML::RARA and t(15;17) translocation, however the HNRNPC::RARG and RARG::HNRNPC fusion transcripts were visualized by RNA sequencing, which showed a poor prognosis to ATO treatment and traditional chemotherapy.

## Case presentation

**Figure 1:**
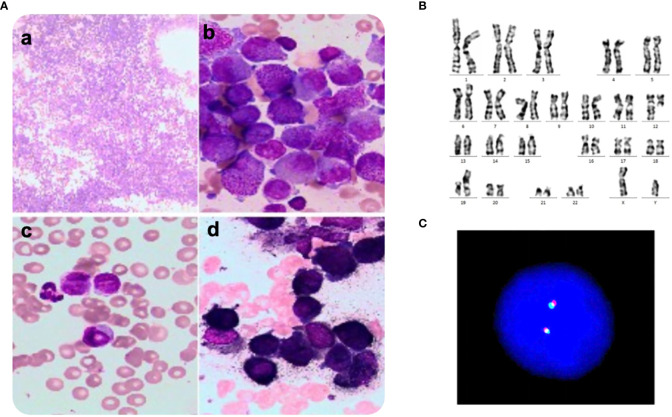
**A**. Morphology, Karyotyping and FISH analysis of bone marrow smear at first consultation. **(A)** Several promyelocytes were shown in the bone marrow aspirate (a, b) and peripheral blood smear (c, d), **(A)** Wright-Giemsa staining, × 100, **(B–D)** Wright-Giemsa staining, × 1000. **(B)** Karyotype of the aberrant clone showing t(12;19)(q13;q13.1). **(C)** Fluorescence in situ hybridization using PML::RARA dual-color, dual-fusion translocation probes and corresponding metaphase chromosomes of the same cell. There was failed to detect the PML::RARA transcript.

**Figure 2:**
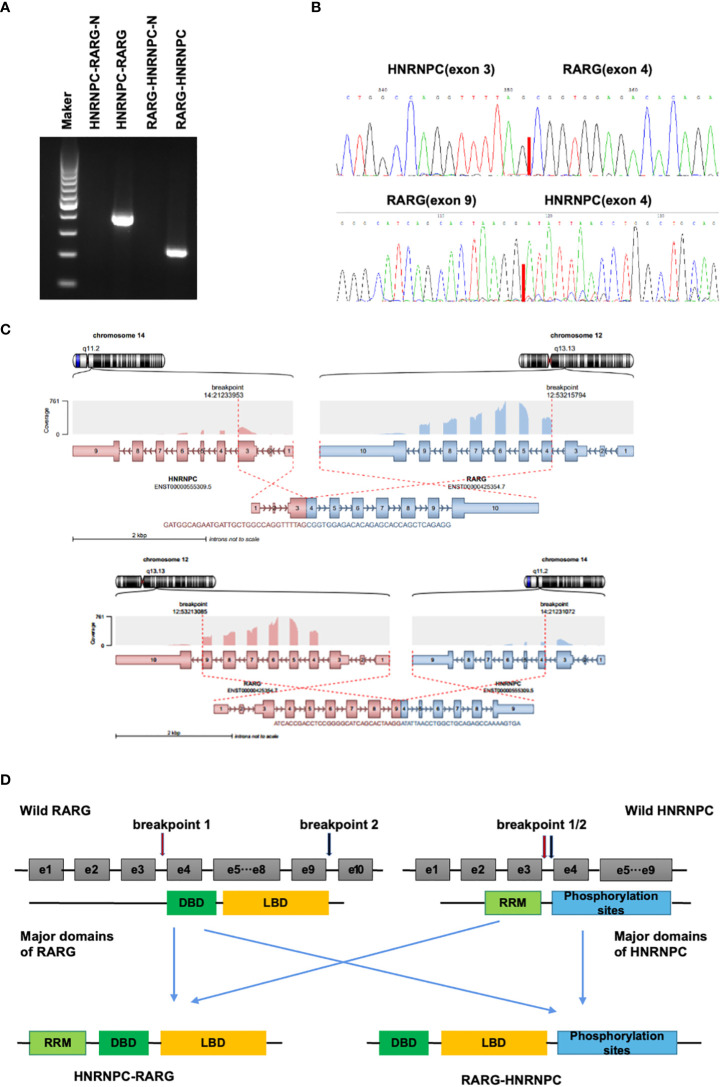
**(A)**. Electrophoresis of reverse transcription-polymerase chain reaction (RT-PCR) products from the patient showed distinct HNRNPC::RARG and RARG::HNRNPC fusion transcripts. **(B)**. Sanger sequencing showing the junction of the HNRNPC::RARG and RARG::HNRNPCP fusion transcripts respectively. **(C)**. The structures of HNRNPC and RARG gene rearrangements and the construction of the corresponding chimeric proteins. **(D)**. Schematic representation of the HNRNPC::RARG and RARG::HNRNPC fusion proteins and its wild-type counterparts. The HNRNPC 3’-region, which contains a cluster of phosphorylation sites, was fused to the RARG 5’-region, which encodes the ligand-binding domain. The HNRNPC 5’-region encodes an RNA recognition motif (RRM), and the segment from RARG encodes a DNA binding domain (DBD).

A 30-year-old man was referred to the local hospital with fever and gingival bleeding for 2 weeks. Peripheral blood test showed white blood cell (WBC) count of 30.35×10^9^/L (10% abnormal promyelocytes), platelet (PLT) count of 94×10^9^/L, and hemoglobin (HGB) level of 110g/L. The results of bone marrow aspirate and immunophenotyping indicated a typical APL. Thus, the patient started the treatment of all-trans retinoic acid (ATRA) (25mg/m^2^/d) immediately, and occurred a suspicious differentiation syndrome (DS) after 3 days of ATRA treatment with symptoms of unexplained fever (≥38°C), progressive weight gain and dyspnea, which was characterized by progressive leukocytosis. Then the patient was transferred to a superior hospital for further treatment. At admission, the peripheral blood test showed WBC count of 41×10^9^/L (65% abnormal promyelocytes), PLT count of 28×10^9^/L, and HGB level of 68g/L. Prothrombin time (PT) was 17.5s (reference, 10.5-13.0s), fibrinogen level was 0.59 g/L (reference, 2.00-4.00 g/L), and D-dimer level was 126.74mg/L (reference, 0.00-0.55 mg/L). Bone marrow aspirates presented markedly myeloproliferative hyperactivity with 80% hypergranular promyelocytes (**Figure 1A a** and **b**), and peripheral blood smear showed POX strong positive with 65% hypergranular promyelocytes (**Figure 1A c** and **d**). Immunophenotyping results showed positive for CD13, CD33, CD117, CD123, CD4, CD9 and CD71, partially expression of CD64 and MPO, but negative for CD34, CD38, HLA-DR, CD11b, CD56, CD7, CD14, CD16, CD19, and CD3. According to the clinical characteristics, BM morphology, and immunophenotype, APL was highly suspected. However, the cytogenetic analysis showed the karyotype of 46, XY, t(12;19)(q13;q13.1) (10), different from the typical reciprocal chromosomal translocation t(15;17)(q24;q21) (10) (**Figure 1B**). And both multiplex polymerase-chain-reaction (PCR) and fluorescence *in situ* hybridization (FISH) failed to detect the typical PML-RARA transcript in the BM sample (**Figure 1C**). In addition, the common fusion genes of APL variants (PLZF::RARA, NPM::RARA, NuMA::RARA, STAT5b::RARA, PRKAR1A::RARA, FIP1L1::RARA, BCOR::RARA, OBFC2A::RARA, TBLR1::RARA, GTF2I::RARA, IRF2BP2::RARA and STAT3::RARA) were undetected. Targeted next-generation sequencing identified KRAS exon2 [NM_033360:c.34G>C(p.G12R)], NRAS exon2 [NM_002524:c.35G>A(p.G12D)] and BCOR exon4 [NM_017745:c.455C>T(p.P152L)] mutations. The diagnosis was considered to be the acute promyelocytic-like leukemias (APLL). Then the patient received arsenic trioxide (ATO 10mg/d) treatment at the first day. Owing to most variant APL exhibiting resistance to ATRA, ATO combined with traditional chemotherapy (Idarubicin and Cytarabine) was adopted as induction therapy. Then we performed a BM aspiration again, which revealed hypercellularity with 50% promyelocytes (**Figure 2**). Thus, the patient received a second induction chemotherapy of Idarubicin, Cytarabine combined with Venetoclax and finally achieved a complete remission in this therapy (**Figure 2**). Subsequently, the patient received three cycles of consolidation chemotherapy, which was combination of Idarubicin, Cytarabine and Venetoclax, the others were intensive treatment of intermediate-dose Cytarabine. Because the patient got an invasive candida infection of liver and lung, and unfortunately the disease relapsed (30% of blasts and abnormal promyelocytes), he failed to receive a hematopoietic stem cell transplantation. The patient underwent a new chemotherapy regimen, consisting of Homoharringtonine, Venetoclax and Azacytidine combination. Unfortunately, the patient died of severe pneumonia in this cycle and the overall survival time less than 10 months.

**Figure 3:**
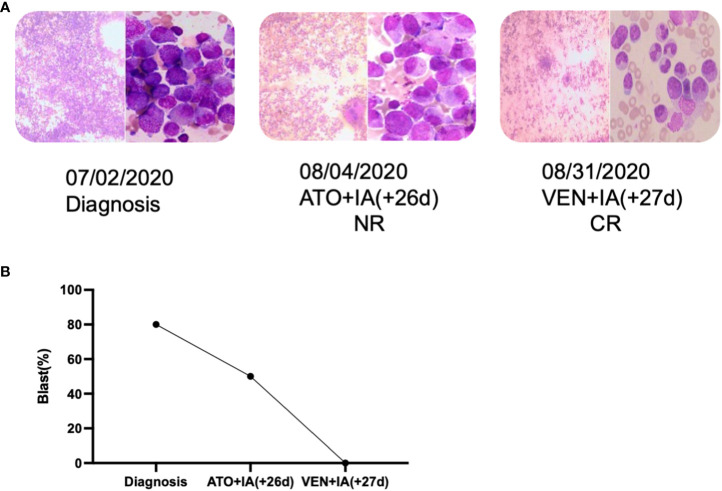
The novel HNRNPC::RARG fusion APLL patient was salvaged by venetoclax combined IA. **(A)** Timeline of induction therapies. **(B)** Bone marrow blast percentages for the patient induction therapy. IA, Idarubicin and Cytarabine; VEN, venetoclax.

Cytogenetic, FISH, and RT-PCR analysis demonstrated the absence of t(15;17)(q24;q21) and PML::RARA in this patient. To characterize the molecular aberrations, we performed RNA sequencing and found a gene fusion event between HNRNPC and RARG. For validation of this gene fusion, we performed RT-PCR and Sanger sequencing to confirm the HNRNPC::RARG fusion transcript in this patient (**Figures 3A, B**). In HNRNPC::RARG, HNRNPC exon 3 was fused in-frame to RARG exon4, and in RARG::HNRNPC, RARG exon 9 was fused in-frame to HNRNPC exon4 (**Figure 3C**). Sanger sequencing demonstrated that the amplicon sequence can be fully aligned with the RNA-seq sequence. The main domains of RARG and HNRNPC were preserved on HNRNPC::RARG fusion protein (**Figure 3D**).

## Discussion

Acute promyelocytic leukemia (APL) is a special subtype of acute myeloid leukemia (AML), more than 90% of the patients achieved excellent outcomes through ATO and ATRA treatment (1). In the previous studies, there are a few cases lacking the PML-RARA fusion transcript while possessing the morphological and immunophenotypic features of typical APL. In this case, we identified a novel fusion transcript of HNRNPC::RARG. The heterogeneous nuclear ribonucleoprotein C (HNRNPC) gene is located at 14q11.2 and encodes hnRNPC protein, which belongs to nuclear RNA-binding protein (6). The functions of HNRNPC are considered to influence pre-mRNA metabolism, including alternative splicing, stabilization, nuclear retention and output of mRNA, and internal ribosome entry site dependent translation (12). The existing studies of HNRNPC mainly focus on solid tumors. Hao, H. et al. found HNRNPC was associated with malignant phenotype and clinical outcomes, as the high levels of HNRNPC indicated poor overall survival (OS) and free of progression (FP) in gastric cancer (GC). And the fundamental study suggested the transfer of HNRNPC location might associate with chemoresistance of GC (12). RARG is a member of the retinoid acid receptor (RAR) family, which modulates the transcription of DNA elements (13). Purton, L. E. et al. demonstrated RARG is a key regulator of the balance between HSC self-renewal and differentiation (14). Several cases harboring a gene rearrangement of RARG such as CPSF6::RARG were reported associated with resistance to ATRA (11). Su Z et al. described an instance of APLL patient with a novel fusion transcript HNRNPC::RARG who was also resistant to ATRA at the time of onset and relapse. The patient then received two cycles of combined chemotherapy prior to CR, and re-induction failed at relapse, indicating that conventional chemotherapeutic protocols are ineffective. Furthermore, a poor prognosis was observed (15). In the report, the HNRNPC and RARG gene break points were the same as in our patient, in HNRNPC::RARG, HNRNPC exon 3 was fused in-frame to RARG exon 4; whereas in RARG::HNRNPC, RARG exon 9 was fused in-frame to HNRNPC exon 4 (15). So until now, there are two cases of HNPNPC::RARG in APL lacking RARA rearrangement in total.

In our study, ATRA was used only for 3 days in this patient because of the incidence of DS, then ATO was adopted instead in combination with the standard 7 + 3 chemotherapy approach, which however was ineffective. How to treat APLL with HNRNPC and RARG rearrangement is tough because of the rare incidence and lacking the detailed biological function. In our study, the patient with HNRNPC and RARG rearrangement had a poor prognosis but achieved remission under the combination of BCL-2 inhibitor (Venetoclax) and standard 7 + 3 chemotherapy. It is the first time that Venetoclax was used to treat a patient with HNRNPC and RARG rearrangement. Our present patient and another reported case with HNRNPC::RARA fusion gene can response effectively to Venetoclax, which suggested its possible role for the patient with APLL (6). It still needs more cases and fundamental experiments to investigate in the future.

In conclusion, we detailed the case with HNRNPC and RARG rearrangement in AML that is similar to APL in morphological and immunophenotypic feature. Nevertheless, the diverse therapy and poor prognosis may indicate a novel subtype of AML, which needs to be confirmed in the future.

## Data availability statement

The datasets presented in this study can be found in online repositories. The names of the repository/repositories and accession number(s) can be found in the article/supplementary material.

## Ethics statement

Written informed consent was obtained from the relevant individual(s), and/or minor(s)’ legal guardian/next of kin, for the publication of any potentially identifiable images or data included in this article.

## Author contributions

WD and LW contributed equally to this study and performed most of the experiments. YG XD, and GW were the principal investigators. ZW, HS, MW, and SC analyzed and discussed the data. All authors contributed to the article and approved the submitted version.
